# Patients’ Perspectives on Stigma of Mental Illness (an Egyptian Study in a Private Hospital)

**DOI:** 10.3389/fpsyt.2014.00166

**Published:** 2014-11-26

**Authors:** Emad Sidhom, Ahmed Abdelfattah, Julie M. Carter, Ahmed El-Dosoky, Mohamed Fakhr El-Islam

**Affiliations:** ^1^The Behman Hospital, Cairo, Egypt

**Keywords:** mental illness, stigma, stigmatization, ostracism, social exclusion, schizophrenia, patient perspectives

## Abstract

The present study is concerned with the stigma of mental illness. It examines the subjective element of the experience of stigma among a sample of in-patients with different mental disorders. The sample was taken from consecutive admissions of in-patients meeting International Classification of Diseases, 10th revision (ICD-10) criteria for mental disorders who had capacity to decide on participation in the study and were willing to respond to the structured interview. The study was undertaken in an Egyptian private psychiatric hospital. The structured clinical interview included aspects of the emotional, behavioral, and cognitive effects of having a psychiatric diagnosis on in-patients with various diagnostic labels in an Egyptian psychiatric hospital. It also studied whether this effect changes with specific disorders, total duration of illness, or sociodemographic variables as gender, age, or educational level. The study illustrated the core items of stigmatization attached to the diagnosis of mental illness (1), which more than half of the participants responded affirmatively. The study aimed to explore the most prevailing aspects of stigma or social disadvantage; hoping that this may offer a preliminary guide for clinicians to address these issues in their practice.

## Introduction

Social stigma can be defined as a state or a set of negative beliefs, disapproval, and discrimination that a society, a tribe, or an ethnic group of people perceives about a socio-culturally unsanctioned state, attitude, or behavior. Those who have contact with people with disabilities are less likely to have negative attitudes (2). Stigma of mental illness has been associated with fear, irresponsibility, and child-like attitudes. It can be subdivided into public and self-stigma (3). The social discrimination against the mentally ill can be historically traced in various aspects as media, policies, literature, and even legally at times. A report from a global program of the World Psychiatric Association (4) suggests that stigma or social disadvantage attaches not only to the mentally ill but also to their families, psychiatric institutions and psychotropic medication. The stigma seems to be particularly harsh in relation to schizophrenia. The report found that in the Egyptian governorate of Ismailia (in the Suez Canal zone) 64% of general practitioners (GPs) thought that patients might become dependent on psychotropic medication and 11% avoided prescribing them. Seventy-five percent of family members of individuals who received the diagnosis of schizophrenia, believed antipsychotic medication led to addiction, 73% of people working in local media said they would probably be afraid to speak to someone with schizophrenia, 66.6% of medical students refused to work with a person known to have a mental disorder, and 81% of secondary school students considered people suffering schizophrenia dangerous. In the same report, results in Egypt were mirrored by those in Morocco, where a majority of individuals with schizophrenia reported stigmatization by neighbors and loss of jobs and friends. Patients in India would not tell neighbors about their mental illness, in order not to jeopardize family members’ chances of getting married, and patients in Canada would not disclose their mental illness in order to be selected for, or avoid the loss of a job. A vicious cycle where by a self-fulfilling prophecy downcasts the mentally ill, their families, and the mental health services. Low esteem by others is internalized by those stigmatized, and the lowered performance associated with low self esteem confirms the initial assumptions about their social inferiority.

The feelings and experiences of 46 people with mental illness were qualitatively studied by narrative interviews by two of the users in community-and day-mental health services in North London (5). Although stigma was a pervasive concern, schizophrenia and drug dependence were the most involved diagnoses, and depression, anxiety, and personality disorders elicited the most patronizing attitudes.

Chamberlin (6) and Thornicroft et al. (7) regretted the rarity of reports on the experiences of people themselves, who have mental illness about the behavior of normal people toward them, and Kadiri (8, 9) regretted the absence of studies of the impact of the psychiatric label in developing countries.

The present study examines the negative effects that could be associated with having a psychiatric diagnosis/label on in-patients in a private practice psychiatric hospital (Behman Hospital) in Cairo, Egypt. It attempts to explore their emotional, behavioral, and cognitive effects of the psychiatric diagnosis/label, and whether the effect changes with specific disorders, total duration of illness and sociodemographic variables. Health system in Egypt relies on both public sector and private sector. Patients who seek the private sector are the ones who can afford to pay for the service. Consequently, they come from a higher socioeconomic status.

## Materials and Methods

The study tries to assess the impact of the social disadvantage (stigma) as internalized by patients having psychiatric diagnosis. The questionnaire used focused on how the patients subjectively internalized the stigma attached to the diagnosis of mental illness and the influence of this on patients’ self-esteem. The questionnaire used in this study tests the patients’ subjectively internalized stigma. The structured interview was prepared by Dr. M. F. El-Islam to inquire about various aspects of stigma. It is composed of 37 item of yes/no questions, to avoid the potential internal inconsistency that can result from the usage of a Likert scale. The choice was due to cultural reasons namely inherent compromised test-retest reliability of assessment tools using Likert-scales with low literate populations (10) as the target population has illiteracy rate of 73.9% (11). The structured clinical questionnaire was not timed, however, retrospectively, each subject required about 20 min to answer the questions. All the questions start with the common prefix “After knowing that you have a psychiatric problem”; where stigma is partly determined by the possible social down-casting related to the emergence of psychiatric disorders. Various items address feelings, thoughts, fantasies, and behavior in a structured interview. Culturally congruent items were selected from other measures of stigma, e.g., Link et al. (12), Phillips et al. (13) and Wahl (14). As compared to the “Survey of Public Attitudes Toward People with Disability,” (2) the survey compared the attitudes of people of were in contact with persons suffering disabilities; to those who do not. It used a diverse questioning style from yes, no, do not know answer to Likert scale answers.

After obtaining the ethical committee’s approval; the study was conducted on in-patients.

**Figure 1:**
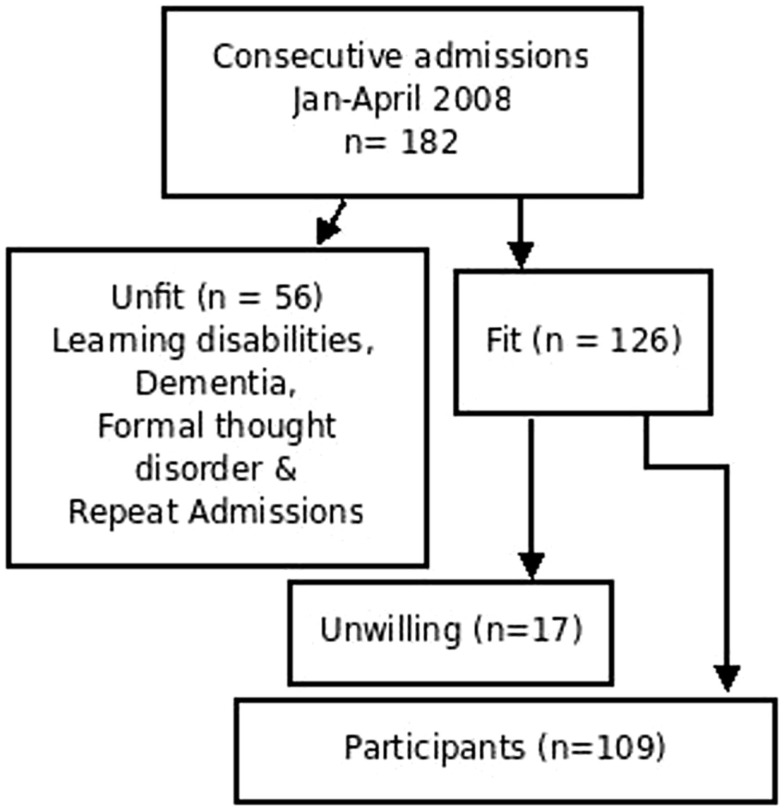
**Sequence of subjects’ recruitment**.

Inter-rater reliability was done by blind ratings of patients simultaneously by the two interviewers, and then collecting data to check the degree of concordance per item. Two interviewers (ES and AA) had inter-rater reliability of 0.91 on the Kappa coefficient for this questionnaire. A pilot of 20 subjects was examined simultaneously to establish the inter-rater reliability. The nature of the yes/no answers of the questionnaire may have contributed to the concordant inter-rater reliability via Cohen–Kappa coefficient. The subjects’ selection included consecutive admissions (*n* = 182) from January 2008 to April 2008 who were willing to participate in the study. Screening for new admissions was performed on daily basis, via reviewing admission records. Exclusion criteria included the diagnoses of organic disorders, learning disabilities and patients suffering from gross thought disorders rendering them unfit to participate (*n* = 56). The exclusion was based on the ICD-10 (15) criteria for organic disorders and learning disabilities and clinical examination for patients suffering formal thought disorders, or too excited to participate in the interview, regardless their diagnosis. An informed consent was obtained from study participants who were willing to participate in the study (*n* = 109) (Figure 1).

One hundred and nine consecutively admitted patients had the structured interview and the data were analyzed using (Ms Julie Carter) SPSS 8.0 for cross tabulation and chi square tests and Open Office Spreadsheet 2.0 for total scores and calculation of mean.

## Results

The mean age of participants was 36.06 years and the mean duration of illness was 5 years. The highest age group was from 26 to 30, followed by 31–35 and 36–40 years old; however, due to a skew in the old age group (from 65 to over 70 years). Fifty seven per cent of the study participants were single and 5% were either divorced, separated, or widowed. The participants were 87 men and 22 women. Women who suffer mental illness are subject to double burden of discrimination (16). The relative under representation of female patients in psychiatric services could be attributed to the protective effect of culture (17). This could be partially attributed to the 25% of the study population that had the diagnosis of substance dependence, where the rate is higher in men than women (18). It could also be correlated to the over-representation of the diagnosis of schizophrenia (44%) where the first admission to psychiatric hospitals is delayed by 3–6 years as compared with males and better clinical outcome on the short-term (19). The excess of males among in-patients in Arab psychiatric in-patients is a general finding and is not limited to the sample studied. However, further research is needed to explore this relative under representation. The expenses of treatments are paid mostly by the families of both male and female in-patients, unless a medical insurance company covers the expenses. The most frequent diagnoses were schizophrenia and related psychoses (*n* = 48), mental and behavioral disorders associated with substance use (*n* = 28), and mood disorders (*n* = 28). Four participants suffered personality disorders as their main diagnosis, and one was multi-symptomatic in the spectrum of anxiety (neurotic) disorders. Items which most participants (>60%) answered affirmatively were considered as the core items of stigmatization ensuing on having a psychiatric diagnosis (Table 1).

**Table 1:** **Core items of stigmatization/disadvantage compared to others**.

1. After knowing that you have a psychiatric problem; do you need faith or traditional healing	89%
2. After knowing that you have a psychiatric problem; do you need to help yourself	85%
3. After knowing that you have a psychiatric problem; do you think others would urge you to consult religious clergy	81%
4. After knowing that you have a psychiatric problem; do you feel sorry for yourself	78%
5. After knowing that you have a psychiatric problem; are you unable to have peace of mind	75%
6. After knowing that you have a psychiatric problem; do you need others’ help	73%
7. After knowing that you have a psychiatric problem; do you feel something is wrong with yourself	72%
8. After knowing that you have a psychiatric problem; are others surprised about your state	68%
9. After knowing that you have a psychiatric problem; have other reduced their contact with you	68%
10. After knowing that you have a psychiatric problem; are you anxious about your future	67%

The question that received the least affirmative answers was about embarrassment from the diagnosis. A preliminary measure was done to compare various groups to each others which is the mean total score. The mean total score of the 37 items interview was 19.83 for all participants. The mean total score for participants who received the diagnosis of mental and behavioral disorder due to substance use was 20.5. The mean total score of participants who received the diagnosis of schizophrenia was 18.44. The mean total score of participants who received the diagnosis of mood disorder was 21.29. The differences in the mean total score as compared to psychiatric diagnoses were not statistically significant.

Pearson correlation, cross tabulation, and Chi square tests were done to correlate the total of each symptom cluster (feelings, thoughts, practice, unpleasant fantasies, and others’ behaviors) of questions with each other. There were significant correlations between most of them.

Cross tabulation, followed by Chi square test was done to find out whether there is significant correlation between sociodemographic variables, diagnoses on one hand and the total of each cluster of items on the other hand. Male gender was correlated with higher change in practice. Younger age correlated with higher feelings of stigmatization and fantasies about others’ reaction. Education was grouped into two brackets; higher and lower education. People with lower education (illiterate/primary education) had many unpleasant fantasies about others’ reactions. Patients who were suffering schizophrenia and related disorders seemed more stigmatized by others’ behavior toward them, and seemed to have more unpleasant fantasies about others’ reactions.

## Discussion

The study results show that the majority affirmed their need to other help besides psychiatric intervention, and that psychiatric labels are not of much significance to them. People who received the diagnoses of schizophrenia and related disorders and mood disorders perceived stigmatization mainly as regards others’ change in behavior toward them, for example, others may get surprised to know they have psychiatric problems, reduce their contact with them, urge them to have faith, urge them to have nothing to do with psychiatrists, or give them fewer responsibilities. These items were also, the ones in their fantasies and expectations from others that significantly correlated with the schizophrenic label. The mean total stigma scores for patients with schizophrenia, substance misuse, and affective disorders were close to each other without any marked lead for schizophrenia.

The study used several questions in order to quantify the responses but cannot claim comprehensiveness. It is limited by the fact that the in-patient groups studied came from very sick patients of moderate to severe psychopathology requiring hospitalization. It would have been helpful to measure the severity of psychopathology using scales like PANSS; but, the scale has not been standardized on Egyptian population. They had higher socioeconomic brackets in order to afford hospitalization in a private hospital. Also, being conducted in a psychiatric facility limited the feasibility of comparison with stigmatization associated with physical illness.

It could be theoretically argued that patients’ statements may be due to the illness. Also, it would have been interesting to measure stigmatization across other groups like organic disorders and learning disabilities; however, competence to give informed consent or participate in the structured interview could make them inaccessible to address these disorders. It would have been also interesting to compare this study with other studies that belong to similar cultural backgrounds, or comparing stigmatization due to mental illness to physical illness within the same culture.

There were some issues addressed by the patients following the interview that could not be quantified during this study, however, they might warrant further research into aspects like the stigma of receiving medication, and the social discrimination due to their side effects as fine tremors, restlessness, tardive symptoms, masked face, shuffling gait or weight gain and the social disapproval of commuting to and from psychiatric hospitals. Further research into the stigma of receiving psychotropic medications and its subsequent impact on adherence might be needed.

## Conflict of Interest Statement

Doctors participating in this study are paid by the patients/study participants in this private hospital. The study was funded by The Behman Hospital.
